# The need for a culturally-tailored gatekeeper training intervention program in preventing suicide among Indigenous peoples: a systematic review

**DOI:** 10.1186/s12888-016-1059-3

**Published:** 2016-10-21

**Authors:** Bushra Farah Nasir, Leanne Hides, Steve Kisely, Geetha Ranmuthugala, Geoffrey C. Nicholson, Emma Black, Neeraj Gill, Srinivas Kondalsamy-Chennakesavan, Maree Toombs

**Affiliations:** 1 Rural Clinical School, School of Medicine, The University of Queensland, Toowoomba, QLD Australia; 2 School of Psychology & Counselling, Queensland University of Technology, Brisbane, QLD Australia; 3 Departments of Psychiatry, Community Health & Epidemiology, Dalhouise University, Halifax, Canada; 4 School of Rural Medicine, University of New England, Armidale, NSW Australia

**Keywords:** Gatekeeper training, Suicide, Indigenous, Suicide prevention, Suicide intervention

## Abstract

**Background:**

Suicide is a leading cause of death among Indigenous youth worldwide. The aim of this literature review was to determine the cultural appropriateness and identify evidence for the effectiveness of current gatekeeper suicide prevention training programs within the international Indigenous community.

**Method:**

Using a systematic strategy, relevant databases and targeted resources were searched using the following terms: ‘suicide’, ‘gatekeeper’, ‘training’, ‘suicide prevention training’, ‘suicide intervention training’ and ‘Indigenous’. Other internationally relevant descriptors for the keyword “Indigenous” (e.g. “Maori”, “First Nations”, “Native American”, “Inuit”, “Metis” and “Aboriginal”) were also used.

**Results:**

Six articles, comprising five studies, met criteria for inclusion; two Australian, two from USA and one Canadian. While pre and post follow up studies reported positive outcomes, this was not confirmed in the single randomised controlled trial identified. However, the randomised controlled trial may have been underpowered and contained participants who were at higher risk of suicide pre-training.

**Conclusion:**

Uncontrolled evidence suggests that gatekeeper training may be a promising suicide intervention in Indigenous communities but needs to be culturally tailored to the target population. Further RCT evidence is required.

## Background

Suicide in traditional Indigenous communities has emerged as a priority issue of international public concern. For instance, until the 1960s, suicide was a rarity in Indigenous communities in Australia [1]. However, the 1970s saw the incidence rates of suicide and suicidal behaviour begin to increase, and by the 1980s, the situation had become endemic in some Australian Indigenous communities [2] and age groups. At present, latest reports indicate that Australian Indigenous men between 25 and 29 still have one of the highest suicide rates in the world [3].

Suicide risk among Indigenous populations is a multifaceted phenomenon, influenced by biological, psychological, and social factors at the individual level, as well as cultural, political, and economic issues at the family and community level [4, 5]. The main risk factors for suicide are mental disorders [6], comorbid physical illness [7], stressful life events as a result of colonisation [7], substance abuse and socioeconomic issues [8].

Many mainstream social risk factors for suicide do not apply to Indigenous peoples in the same way given their different social structures; Indigenous concepts surrounding suicide differ from Western concepts [9–11]. Consequently, the need to develop a culturally appropriate and effective suicide prevention program, that will be accepted and effective for Indigenous people, is essential. Many risk factors occur at disproportionately high rates in Indigenous populations, placing them at significantly higher risk of suicide than the general population [7]. For instance, Indigenous people are more likely than the general population to use alcohol and drugs at levels that increase their risk of mental disorders [12]. Furthermore, higher levels of social disadvantage such as unemployment, homelessness, and incarceration increase their exposure to stressful life events, which, in turn, increase the risk of suicide [12]. As a result, a culturally acceptable suicide prevention training program may be more effective in reducing the risk of suicide for Indigenous populations.

### Suicide prevention

In 1996, the United Nations formulated official guidelines for national suicide prevention strategies that encouraged governments to adopt comprehensive approaches [13]. Primary prevention can either focus on an entire population or on high-risk groups. Most suicide prevention strategies involve either reducing risk factors for suicide or seeking out people at risk for referral and eventual treatment [14]. The ultimate aim would be to reach those at risk who are outside the scope of healthcare and health professionals [15]. For example, broad psychological education programs aimed at school-age people in general, as well as specialised programs targeting specific populations have been proposed as potentially effective strategies for reducing suicide rates [15]. Case-finding strategies include general education, specific care-provider screening programs, and gatekeeper training [16]. Gatekeeper training, in particular, has emerged as a promising suicide prevention initiative and has now received support worldwide [14].

### Gatekeeper training

Gatekeepers are usually people who provide access to something for someone in need. Gatekeeper suicide intervention training, which dates back to the late 1960s, teaches specific groups of people to identify others at high risk for suicide and refer them to treatment [14]. In essence, gatekeepers *open the gate to help* for people at risk of suicide. Historically, gatekeepers have been divided into two main groups, designated or emergent. Designated groups include professionals such as those in medicine, social work, nursing and psychology. Emergent groups are community members who may not have been formally trained in suicide prevention, but who may have contact with people who have suicidal intent. These may include recreation staff, police, coaches, teachers, counsellors and community service providers. It has also been suggested that family, friends, and peer-helpers may be appropriate given their close relationships with those at risk for suicide [14]. Gatekeeper training may be particularly appropriate for those who value the importance of relationships within their community.

A previous systematic review of suicide prevention programs, restricted to Indigenous peoples from Australia, United States, Canada and New Zealand [12] identified four studies on gatekeeper training. These studies identified short-term gains as outcomes, and were limited to participants’ knowledge and confidence in how to identify individuals at risk of suicide, and their intention to help those at risk of suicide. No randomised controlled trials (RCTs) were identified. The aim of the current paper was to conduct a revised systematic review of gatekeeper training programs targeting Indigenous peoples in any country, to determine the effectiveness and cultural appropriateness of gatekeeper training for Indigenous people.

## Methods

### Search strategy

A systematic literature search was undertaken, informed by guidelines from the Preferred Reporting Items for Systematic Reviews and Meta-Analysis model (PRISMA) [17] by the main author (BN). Fifteen databases (Table 1) were explored using the keyword “suicide”, suicide prevention”, “suicide intervention”, “gatekeeper” and “training” in article titles and abstracts. The article titles and abstracts were then screened for the keyword “Indigenous” and other internationally relevant alternatives: “Maori”, “First Nations”, “Native American”, “Alaskan Native”, “Inuit”, “Metis” and “Aboriginal”. The database search was supplemented by a separate search of resources from three more targeted sites: Australian Bureau of Statistics, Australian Institute of Health and Welfare, and Cochrane Library.

**Table 1:** List of databases and targeted sites searched for review

Databases
• Australian Medical Index
• Australian Public Affairs Information Service – Health
• Aboriginal and Torres Strait Islander subset Health
• Health & Society
• Rural and Remote Health Database
• Indigenous Studies Bibliography
• Indigenous Australia
• Family-Aboriginal and Torres Strait Islander Subset
• Far North Queensland Collection
• Informit Indigenous Collection
• Australian Library and Information Science Abstracts
• ProQuest Research Library
• MedLine
• EMBASE
• Psych Info
Targeted Searches
• Australian Bureau of Statistics
• Australian Institute of Health and Welfare
• Cochrane Library

### Inclusion criteria

The review included articles published in English between the years 2000 and 2016 on gatekeeper training programs that targeted Indigenous populations in any country. This review specifically looks at ‘gatekeeper training’; thus no attempt to include other suicide intervention or prevention programs was made.

### Data extraction and validity assessment

**Fig. 1:**
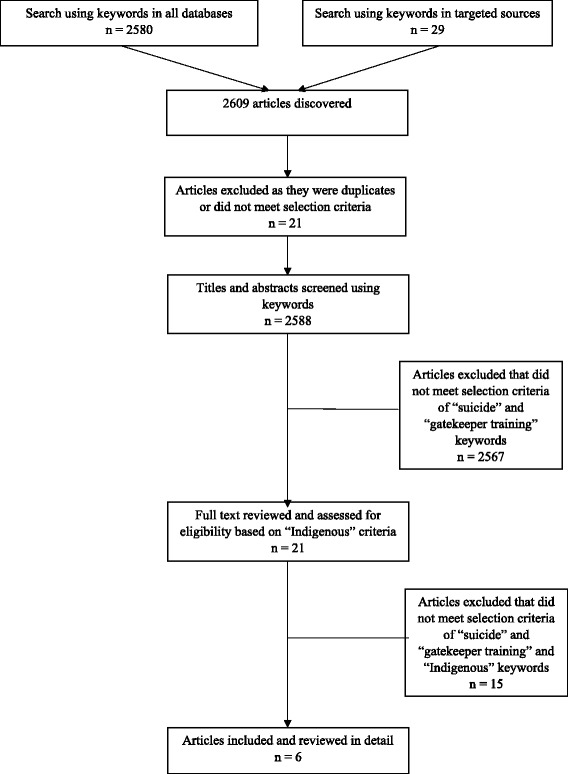
Flow chart of literature search strategy

The main reviewer (BN), examined titles and abstracts against the eligibility criteria. Following exclusion of duplicates (Fig. 1), full text articles of the remaining potentially relevant articles were extracted and reviewed in detail by the main reviewer. Articles deemed as suitable according to the selection criteria were cross-checked and re-read by co-authors (MT and SKC) (Fig. 1). We followed the methodological framework for scoping studies described by Arksey and O’Malley [18], and further elaborated by Levac et al. [19] to design our methodology.

### Analysis

Due to the small number of studies identified and the wide range of methodologies and outcomes used, we undertook a systematic review using narrative synthesis, and made no attempt to meta-analyse the data. Due to the heterogeneity of the studies, a textual narrative of the study characteristics was used for narrative synthesis analysis, and producing study outcomes by all co-authors.

## Results

### Search results

The keyword “suicide”, “suicide prevention”, and “suicide intervention” identified 2609 articles (Fig. 1). Removing duplicates and those that did not meet the selection criteria, left 2588 articles. These 2588 articles where then screened for the keywords “gatekeeper training”, and “Indigenous” and its other descriptors, leaving 21 potentially relevant articles that were reviewed for inclusion. Of these, six articles, comprising five studies, focused on gatekeeper training for Indigenous communities. A summary of the studies and their findings is described in Table 2.

**Table 2:** Characteristics of the evaluations of gatekeeper training programs targeting Indigenous individuals

Study	Population	Study design	Training details	Gatekeeper participants	Aims	Summary of outcome measures
Capp et al. 2001 [20]	Australian Indigenous Youth	Self-reported, baseline with follow-up investigation	Workshops	Community members, professionals and university students *N* = 44 (F = 40, M = 4) Mean age = 36 years	Reduce youth suicide through increased ability to identify at risk individuals, and refer to professional help	Significant increase in knowledge, increase in self-efficacy, no changes in behavioural intentions, significant decrease in intention to refer to medical services
Deane et al. 2006 [21]	Australian Indigenous Youth	Self-reported, baseline and follow-up investigation	Workshops	*N* = 40 (91 % follow-up) community members	Identify long term effects of inclination to perform gatekeeper training behaviours as a result of the intervention program	The increase in helping at risk people, intentions to help and confidence to identify at risk individuals was sustained at 2 years follow-up. Significant relationship between intentions to help prior to workshop and actually helping someone at risk was witnessed.
Westerman 2007 [22]	Youth & Australian Indigenous Community members	Pre/post training questionnaires	Workshops	Indigenous Psychological Services *N* = 997 to date *N* = 769 follow-up	Train mental health service providers to target at risk individuals and prevent suicide	Increase in skills, confidence, intention to help, intention to refer to professional, and better understanding of ‘cultural myths’ of suicidal behaviour
Muehlenkamp et al. 2009 [23]	Native American College students	Assess knowledge scores	Workshops	*N* = 90 AI^a^ students, ‘Medicine Wheel’ & QPR^b^	Increase AI^a^ suicide prevention program use	Reported high levels of satisfaction post study, increased knowledge and student learning
Lafromboise and Lewis 2008 [24]	Zuni Native American youth	School-based skills training	Curriculum based	School-based employees, *N* = 128	Increase cultural awareness and acceptance of need for suicide prevention	Positive impact on hopelessness, suicidal ideation and students’ abilities to intervene in a peer suicidal crisis situation
Sareen et al. 2013 [26]	Canadian First Nations members, age <16 years old	Self-reported, Case/Control analysis	2 days of ASIST^c^ training vs 2 days of Resilience Retreat (control)	RCT: *N* = 31 ASIST^c^ participants *N* = 24 Resilience retreat control participants	Investigate a controlled evaluation of ASIST^c^ gatekeeper training	Based on ITT^d^ analysis, a significant impact on suicide intervention capabilities was not seen between those on the Retreat and those provided ASIST^c^ training. Trends towards increased self-reported suicidal ideation for those on ASIST^c^ training.

^a^ *AI* Native American, ^b^ *QPR* question, persuade, and refer, ^c^ *ASIST* applied suicide intervention skills training, ^d^ *ITT* intention to treat. RCT

### Study characteristics

The five gatekeeper training studies were conducted within Indigenous communities in Australia (*n* = 2), Canada (*n* = 1) and the USA (*n* = 2), within the past 16 years. Four were uncontrolled pre- and post-training studies and the fifth was an RCT. A pre- and post-training study from Australia evaluated a community-based gatekeeper program targeting Indigenous youth in regional New South Wales [20] to reduce youth suicide through increased ability to identify at risk individuals, and refer to professional help (Table 2). These participants were followed up 2 years later [21] to identify long term effects of the intervention training provided. A second Australian study investigated Indigenous suicide prevention programs delivered in Western Australia by Indigenous Psychological Services using gatekeeper training skills [22] for mental health service providers to identify at risk individuals and prevent suicide. Two studies implemented a school or college intervention in the USA [23, 24] and provided increased awareness and acceptance for suicide prevention training programs. The sole RCT investigated a controlled gatekeeper training evaluation of Applied Suicide Intervention Skills Training (ASIST) [25] in a Canadian First Nations community [26]. Four of the five studies used workshops to train their gatekeepers, and one (from the USA) was curriculum-based within an educational setting. Only one study [24] aimed to increase cultural awareness by developing a culturally informed and tailored intervention model.

### Study outcomes

The four uncontrolled gatekeeper training studies reported a range of positive findings (Table 2). Capp et al. [20] found a significant increase in knowledge and self-efficacy, but no changes in suicidal behavioural intentions, which were already high at baseline. A significant decrease in intentions to refer to medical services was also seen. A 2 year follow up of 91 % (*n* = 40) of participants found that the increase in intentions to provide help was sustained, with 15 (37.5 %) participants reporting they had helped someone at risk of suicide after completing training. The results also indicated there was a significant relationship between participants’ pre-workshop confidence and intentions to help, and actually helping somebody who was suicidal [21]. Westerman [22] identified an increase in skills, confidence, and intentions to help or refer to a professional, as well as a better understanding of ‘cultural myths’ of suicidal behaviour post training. Muehlenkamp [23] reported high levels of satisfaction after the intervention, and increased knowledge. The final pre- and post-training study described a positive impact on participants’ levels of hopelessness, suicidal ideation and abilities to intervene in a peer suicidal crisis situation [24]. This study was also the only study to increase cultural awareness and acceptance for suicide prevention [24].

The quality of the four included pre- and post-training studies was not ideal; only one examined medium term outcomes by following participants over a 2-year period. Retention was high (91 %) and the positive gains were maintained over a period of 2 years [20, 21]. Three studies relied on self-report measures, developed specifically to assess the outcomes of the study with no evidence for their reliability or validity [20, 22, 24]. The sole RCT differed from the other studies in that the participants were Canadian First Nations members aged 16 years or younger, who were at risk of suicidal ideation or attempt at pre-training [26]. At baseline, there were no significant differences in adolescent distress levels between those who received gatekeeper ASIST training [25] and those in a control group (who participated in a resilience retreat). Subsequently, people who underwent gatekeeper training in this RCT were not more likely to engage in gatekeeper behaviours over the 6-month follow up period in comparison to those in the control training group. In addition, there was a trend towards greater harm, and increased suicidal ideation in those receiving gatekeeper training [26]. Although this study had the most robust design and used an intent to treat analysis, it had a small sample size (*n* = 55). Participants also had a history of suicidal ideation and/or attempts at baseline, and may not have been suitable for becoming resilient gatekeepers. Although the adolescents were randomised to the treatment *versus* control group using a random number generator, the gatekeeper-trained group had significantly lower levels of education than the control group. It is also unclear whether the assessors were blinded to the group allocation at follow up and only 50 (91 %) of the initial 55 subjects completed the 6 month follow up [26]. The ASIST [25] program is a general suicide prevention program, with benefits seen only for short interventions, and may not have been the most suitable program for gatekeeper training in this 6 month follow-up study [27]. In addition, the program was not culturally modified in any way. Nevertheless, the primary outcome was rated using the Suicide Intervention Response Inventory [28], a validated instrument that is sensitive to changes following gatekeeper training.

## Discussion

The present paper updates a systematic review conducted four years ago on gatekeeper training among Indigenous communities, by specifically analysing the cultural appropriateness of training programs, besides also determining their effectiveness. Furthermore, two additional studies were identified, including one RCT. Unlike the previous review, we did not restrict our search to Indigenous peoples from Australia, United States, Canada and New Zealand. In spite of this, all the included studies came from three of those four countries. Support for the effectiveness of gatekeeper suicide intervention training within Indigenous communities was found in the four uncontrolled studies reviewed. However, beneficial effects were largely restricted to changes in knowledge or attitudes, rather than behaviour. These findings were not confirmed in the only RCT conducted to date, which found no significant differences between the gatekeeper training group and controls on intention to perform gatekeeper behaviours. The RCT used an internationally accepted suicide prevention training program (ASIST) but without cultural modification. Importantly, no study evaluated any effect on suicide attempts, and only one study aimed to increase cultural awareness by developing a culturally informed and tailored intervention model.

### Indigenous suicide prevention though gatekeeper training

Gatekeeper training has some inherent strengths. The training can be moulded to address specific issues that arise in different regions. This could include training for communities affected by cluster suicides, or using local statistics on substance use to highlight specific local risk factors. In addition, training familiar community members (rather than outsiders) uses existing relationships to help those at risk, and avoids the onerous and difficult task of creating new pathways to care. Importantly, gatekeepers receive education in an area that ultimately strengthens their respective environments, helping them to take control of situations in which they previously may have felt helpless [14, 16].

However, there may also be practical barriers to the implementation of gatekeeper training in Indigenous communities. Community members need to be interested and invested in recognising the need for suicide prevention [14]. A wider range of strategies need to be available. In addition, people at high risk for suicide should understand why referral and treatment is necessary. Many people in smaller communities, both in urban and rural areas, may have significant concerns regarding confidentiality, privacy, and trust [20]. There is also the potential that people referred to treatment may not be willing to accept help if it is from professional mental health care staff, owing to the stigma that may exist in using these services [20]. Finally, there is conflicting evidence on whether the effect of gatekeeper training tapers off over time [4, 29]. A qualitative study of gatekeepers indicated that top-up interventions may help sustain the effects of gatekeeper training [29].

### Study limitations

The main limitation of this review is that only five studies were identified, and only one was an RCT. The studies were restricted to just three countries and predominantly in North America, limiting the generalizability of results. It is possible that the sole RCT included may have been underpowered to detect statistically significant results. Worryingly, it showed a trend to increased suicidal ideation in participants receiving the training, which might suggest that intervention may lead to possible harms. The subjects in the RCT were themselves, at a higher risk of suicide, suggesting that it may be necessary to screen participants prior to gatekeeper training to minimise such risk. In addition, beneficial effects in all the included studies, where present, were largely restricted to changes in knowledge or attitudes rather than behaviour.

### Implications

Suicide prevention strategies targeting Indigenous suicide often use frameworks that are based on non-Indigenous understandings of suicide. With suicide being a seemingly recent phenomenon for Indigenous peoples, there is limited understanding about specific risk factors and how to best respond to suicide risk in this population, as well as a scarcity of Indigenous specific suicide prevention resources or services [30].

The studies analysed within this review offer limited support for the effectiveness of training community members in the recognition of individuals at risk of suicidal behaviour using current models of suicide intervention and prevention [30]. These largely uncontrolled findings suggest that: (a) gatekeeper programs may hold promise for creating a community safety net, and (b) there is a lack of culturally appropriate programs, that are specific for Indigenous people, and can evaluate gatekeeper knowledge and skills.

Further research using controlled, systematic and culturally appropriate intervention training methods is required in order to evaluate effectiveness of gatekeeper training in Indigenous communities as a stand-alone intervention and also as a part of a comprehensive suicide prevention strategy. These should utilise suitable control conditions, and evaluate a range of outcome measures, including independent evaluations of risk assessment and management skills as well as changes in knowledge, confidence, and intentions to help people at risk of suicide. Changes in helping behaviour including referral and treatment should also be assessed [16]; and a wider implementation of the training programs should also be evaluated.

## Conclusion

Uncontrolled evidence suggests that gatekeeper training may be a promising suicide intervention in Indigenous communities but this needs to be tailored to the target population. Further RCT evidence is required to determine the effectiveness of suicide prevention gatekeeper training programs that are culturally appropriate for Indigenous populations. The development of culturally-tailored and effective suicide prevention programmes specifically for Indigenous people are therefore essential.
